# Supplementary material for: Patterns of Care and Data Quality in a National Registry of Black and White Patients with Merkel Cell Carcinoma
Source: Cancers (Basel). 2022 Oct 15;14(20):5059. doi: 10.3390/cancers14205059 (PMC9600131; doi:10.3390/cancers14205059)
Supplement: Supplementary file 1 [file cancers-14-05059-s001.zip › cancers-1900890-supplementary.pdf]

Supplementary Materials: Patterns of Care and Data Quality in a National Registry of Black and White Patients with Merkel Cell Carcinoma

Abbas Rattani, Jeremy Gaskins, Grant McKenzie, Virginia Kate Scharf, Kristy Broman, Maria Pisu, Ashley Holder, Neal Dunlap, David Schwartz and Mehran B. Yusuf

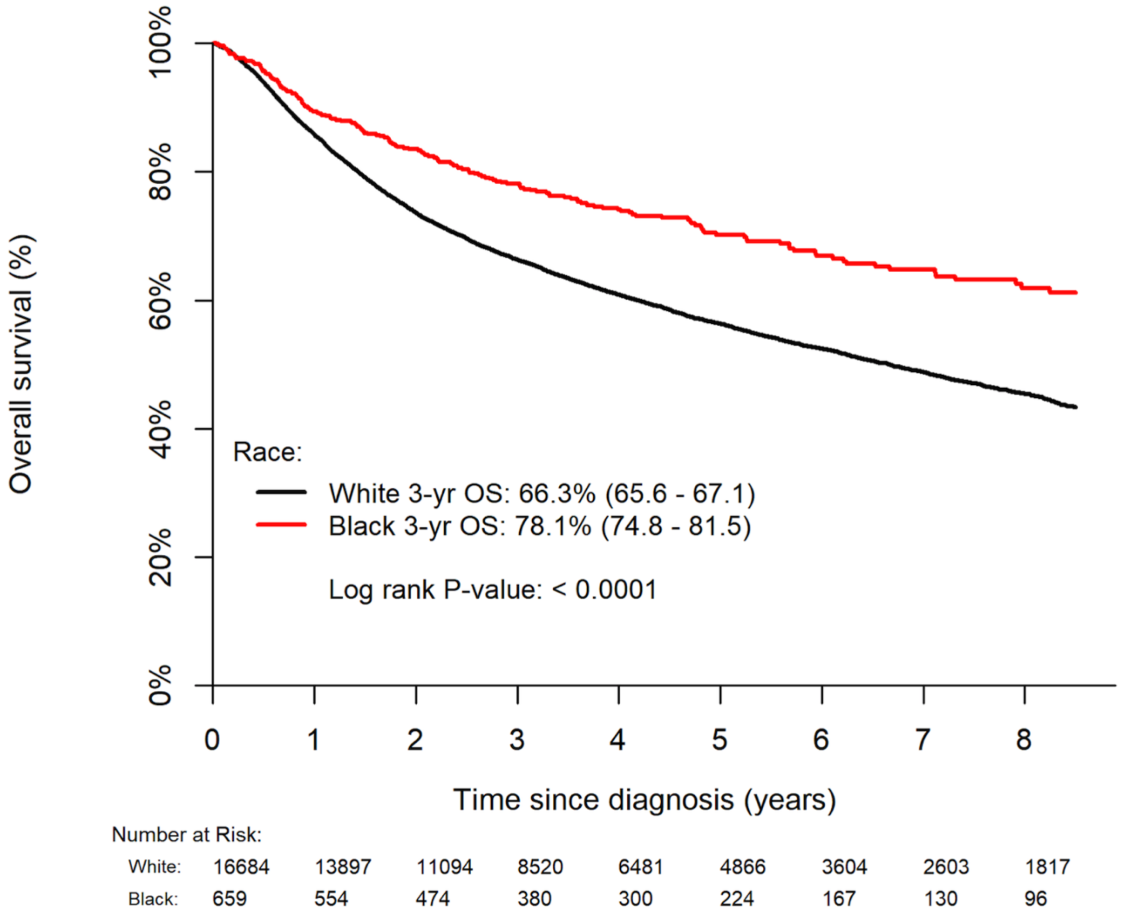

Figure S1. Kaplan Meier Curve for Overall Survival by Race for Patients with Known Staging

Table S1. Cohort characteristics by race in the sensitivity analysis cohort (known stage only).

		Total		White		Black		
		**N**	**Prop**	**N**	**Prop**	**N**	**Prop**	**P-value**
		19503		18769	96%	734	4%	
**Age**								***<0.0001***
	49 or less	1121	6%	979	5%	142	19%	
	50-59	2111	11%	1958	10%	153	21%	
	60-69	4301	22%	4118	22%	183	25%	
	70-79	5885	30%	5718	30%	167	23%	
	80+	6085	31%	5996	32%	89	12%	
**Sex**								***<0.0001***
	Male	11929	61%	11591	62%	338	46%	
	Female	7574	39%	7178	38%	396	54%	
**Insurance Status**								***<0.0001***
	Private Insurance	5280	27%	5101.7	27%	261.9	36%	
	Not Insured	244	1%	216	1%	32.7	4%	
	Government	13712	71%	13451.3	72%	439.4	60%	
	Unknown	267						
**Analytic Stage**								***<0.0001***
	Stage I	9555	49%	9216	49%	339	46%	
	Stage II	3914	20%	3688	20%	226	31%	
	Stage III	4602	24%	4514	24%	88	12%	
	Stage IV	1432	7%	1351	7%	81	11%	
**Definitive Resection**								***0.0146***
	No Definitive Resection	9859	51%	9455	50%	404	55%	
	Definitive Resection	9644	49%	9314	50%	330	45%	
**Procedure Type**								***0.0428***
	No Procedure	1889	10%	1821	10%	68	9%	
	Excision/Biopsy/Other	7970	41%	7634	41%	336	46%	
	Mohs	1621	8%	1570	8%	51	7%	
	Wide Local Excision	8023	41%	7744	41%	279	38%	
**Definitive Resection (within first 42 days)**								***<0.0001***
	No Definitive Resection	13020	67%	12459	66%	561	76%	
	Definitive Resection	6483	33%	6310	34%	173	24%	
**Time to Definitive Resection Quartile**								***<0.0001***
	Within 21 days	2723	28%	2650	28%	73	22%	
	22 - 35 days	2703	28%	2631	28%	72	22%	
	36 - 56 days	2414	25%	2341	25%	73	22%	
	Greater than 57 days	1804	19%	1692	18%	112	34%	
	No Definitive Resection	9859		9455		404		
**Radiation**								***<0.0001***
	No Radiation	11305	58%	10742	57%	563	77%	
	Radiation	8198	42%	8027	43%	171	23%	
**Time to Radiation Quartile**								***<0.0001***
	Within 60 days	2153	26%	2118	26%	35	20%	
	61 - 80 days	1929	24%	1898	24%	31	18%	
	81 - 110 days	2144	26%	2112	26%	32	19%	
	Greater than 110 days	1972	24%	1899	24%	73	43%	
	No RT	11305		10742		563		
**Chemotherapy**								0.7670
	No Chemo	17756	91%	17085	91%	671	91%	
	Chemotherapy	1747	9%	1684	9%	63	9%	
**Time to Chemo Quartile**								0.8991

	Within 30 days	360	21%	349	21%	11	17%	
	31 – 60 days	464	27%	446	26%	18	29%	
	61 – 100 days	465	27%	449	27%	16	25%	
	Greater than 100 days	458	26%	440	26%	18	29%	
	No Chemo	17756		17085		671		
**Charlson/Deyo Comorbidity Score**								***0.0194***
	0	14637	75%	14113	75%	524	71%	
	1	3328	17%	3194	17%	134	18%	
	2 or more	1538	8%	1462	8%	76	10%	
**Distance to Treatment Center**								***<0.0001***
	Less than 10 mi	7652	44%	7900.5	42%	475.1	65%	
	10-25 mi	4842	28%	5263.4	28%	146.4	20%	
	25-50 mi	2400	14%	2677.2	14%	59.4	8%	
	50-100 mi	1544	9%	1742.5	9%	37	5%	
	100 mi or more	1046	6%	1185.4	6%	16.1	2%	
	Unknown	2019						
**Zip Code Income Level**								***<0.0001***
	$63,000+	6519	37%	7094.4	38%	152	21%	
	$48,000-63,000	4858	28%	5273.7	28%	158.8	22%	
	$38,000-48,000	3890	22%	4180.8	22%	172.8	24%	
	$0-38,000	2193	13%	2220.1	12%	250.4	34%	
	Unknown	2043						
**Diagnosis Year**								0.9258
	2006 – 2008	3042	16%	2924	16%	118	16%	
	2009 – 2011	4323	22%	4164	22%	159	22%	
	2012 – 2014	5540	28%	5326	28%	214	29%	
	2015 – 2017	6598	34%	6355	34%	243	33%	
**Tumor Size**								***<0.0001***
	No mass found/Microscopic	421	3%	805.1	4%	21.5	3%	
	Less than 2cm	8131	62%	11597.5	62%	384.4	52%	
	2-5cm	3651	28%	5028.4	27%	243.7	33%	
	Greater than 5cm	1004	8%	1338	7%	84.4	11%	
	Size unknown	6296						
**Resection Margins**								0.5032
	No Residual Tumor	15069	89%	14967.2	88%	581.8	87%	
	Residual Tumor	1936	11%	1980.8	12%	84.2	13%	
	No Definitive Resection	1889		1821		68		
	Definitive Resection w/unknown margins	609						
**Primary Site**								***<0.0001***
	Head/Neck	8974	46%	8654	46%	320	44%	
	Trunk	2810	14%	2652	14%	158	22%	
	Limbs	6658	34%	6432	34%	226	31%	
	Other/NOS	1061	5%	1031	5%	30	4%	
**Immune Suppression**								0.0517
	Immunocompetent	5223	89%	16352	87%	548.6	75%	
	Immunosuppressed	631	11%	2417	13%	185.4	25%	
	Unknown	13630						
**Positive Lymph Nodes**								***0.0125***
	None positive	5702	58%	12821.8	68%	565.7	77%	
	1-3 positive	3229	33%	4679.6	25%	131	18%	
	4-8 positive	544	6%	771	4%	22.9	3%	
	9 or more positive	344	4%	496.6	3%	14.4	2%	

	Unknown	9684						
**Facility Type**								***<0.0001***
	Academic/Research Program	9197	48%	9010.9	48%	415.2	57%	
	Community Cancer Program	7838	41%	7717.1	41%	216.7	30%	
	Integrated Network Program	2101	11%	2041	11%	102.1	14%	
	Facility Type Suppressed (age<40)[a]	367						
**Facility Case Volume**								0.0686
	Lowest Third	6403	33%	6174	33%	229	31%	
	Middle Third	6409	33%	6139	33%	270	37%	
	Largest Third	6691	34%	6456	34%	235	32%	

The cell counts and percentage values stratified by race are based on the imputed datasets, as this better represents the population distribution for these factors. Any sets that do not add to 100% comes from rounding each percentage to whole numbers.

Chemo: chemotherapy
cm: centimeter
mi: mile(s)
NOS: Not otherwise specified
RT: Radiation Therapy

[a] To ensure patient privacy, NCDB suppressed facility type for all patients with age < 40. For these patients, facility is included in the multiple imputation step as with all other missing data.

Table S2: Race effects from multivariate modelling of other clinical outcomes in the sensitivity analysis cohort (known stage only).

	Race Effect	Confidence Interval	p-value
Disease Characteristics			
Cancer Stage[A1]	1.01	0.87 – 1.17	0.8973
Tumor Size[A1]	1.42	1.19 – 1.69	***0.0001***
Number of Positive Nodes[A1]	0.72	0.54 – 0.95	***0.0287***
Subsite[B2]			***0.0001***
Head/Neck	ref		
Trunk	1.19	0.96 – 1.47	0.1049
Limbs	0.75	0.63 – 0.90	***0.0023***
Other/NOS	0.72	0.49 – 1.07	0.1022
Treatment Characteristics			
Facility Volume[A3]	1.59	1.37 – 1.84	***<0.0001***
Definitive Resection (within 42 days)[C4]	0.64	0.53 – 0.76	***<0.0001***
Time to Definitive Resection Quartile[A4*]	1.78	1.43 – 2.21	***<0.0001***
Positive Surgical Margins (DR pts only)[C4]	0.67	0.38 – 1.20	0.1796
Radiation (DR pts only)[C4]	0.55	0.41 - 0.72	***<0.0001***
Time to Radiation Quartile (DR pts onlycategorical)[A4*]	1.74	1.11 – 2.72	***0.0149***
Radiation (no DR pts only)[C4]	0.44	0.34 - 0.56	***<0.0001***
Time to Radiation Quartile (no DR pts only)[A4*]	1.83	1.23 – 2.73	***0.0029***
Chemo (DR pts only)[C5]	0.89	0.50 - 1.59	0.6983
Time to Chemo Quartile (DR pts only)[A5*]	2.85	0.88 – 9.27	0.0811
Chemo (no DR pts only)[C5]	0.88	0.59 – 1.30	0.5092
Time to Chemo Quartile (no DR pts only)[A5*]	1.04	0.61 – 1.79	0.8751
Data Quality			
Missing Cancer Stage[C6]	NA		
Missing Tumor Size[C6]	0.96	0.78 - 1.17	0.6720
Missing Nodal Information[C6]	1.80	1.49 - 2.18	***<0.0001***

The model implemented depends on the type of outcome variable considered:
A. Ordinal logistic regression for ordinal outcomes (race effect is the proportional odds ratio)
B. Baseline categorical logistic regression for unordered categorical outcomes (race effect is the odds ratio relative to ref baseline)
C. Binary logistic regression for binary outcomes (race effect is odds ratio)
*Represents a model only among patients who receive that treatment

The set of confounders for each model was chosen a priori based on clinical relevance. The included confounders are the following:

1. Confounders: age, sex, insurance, distance to treatment facility, Charlson/Deyo comorbidity score, diagnosis year, zip code income, subsite, immunosuppression
2. Confounders: age, sex, insurance, distance to treatment facility, Charlson/Deyo comorbidity score, diagnosis year, zip code income, immunosuppression

3. Confounders: age, sex, insurance, distance to treatment facility, Charlson/Deyo comorbidity score, diagnosis year, zip code income, subsite, immunosuppression, stage, tumor size
4. Confounders: age, sex, insurance, distance to treatment facility, Charlson/Deyo comorbidity score, diagnosis year, zip code income, subsite, immunosuppression, stage, tumor size, facility volume, facility type
5. Confounders: age, sex, insurance, distance to treatment facility, Charlson/Deyo comorbidity score, diagnosis year, zip code income, subsite, immunosuppression, stage, tumor size, facility volume, facility type, radiation
6. Confounders: age, sex, insurance, distance to treatment facility, Charlson/Deyo comorbidity score, diagnosis year, zip code income, subsite, immunosuppression, stage, tumor size, facility volume, facility type, radiation, chemotherapy (within 90 day)

Chemo: Chemotherapy
DR: Definitive Resection
pts: patients
RT: Radiation Therapy
NOS: Not Otherwise Specified

Table S3: Cox regression modeling for overall survival in the sensitivity analysis cohort (known stage only).

	Adj HR	95% CI			P-values
**Race**				***0.0001***	
White	Reference				
Black	0.74	0.63	0.86		***0.0001***
**Age**				***<0.0001***	
49 or less	Reference				
50-59	1.81	1.48	2.21		***<0.0001***
60-69	2.26	1.88	2.72		***<0.0001***
70-79	3.52	2.92	4.24		***<0.0001***
80+	7.15	5.93	8.61		***<0.0001***
**Sex**				***<0.0001***	
Male	Reference				
Female	0.75	0.71	0.79		***<0.0001***
**Insurance Status**				***<0.0001***	
Private Insurance	Reference				
Not Insured	1.36	1.07	1.73		***0.0108***
Government	1.29	1.20	1.39		***<0.0001***
**Analytic Stage**				***<0.0001***	
Stage I	Reference				
Stage II	1.20	1.08	1.33		***0.0007***
Stage III	1.43	1.22	1.69		***<0.0001***
Stage IV	2.83	2.39	3.34		***<0.0001***
**Procedure (time-varying covariate)**				***<0.0001***	
No Procedure	Reference				
Excision/Biopsy/Other	0.67	0.61	0.74		***<0.0001***
Mohs	0.61	0.54	0.69		***<0.0001***
Wide Local Excision	0.64	0.58	0.70		***<0.0001***
**Radiation (time-varying covariate)**				***0.0023***	
No Radiation	Reference				
Radiation	0.92	0.88	0.97		***0.0023***
**Chemotherapy (time-varying covariate)**				***<0.0001***	
No Chemo	Reference				
Chemotherapy	1.36	1.26	1.47		***<0.0001***
**Charlson/Deyo Comorbidity Score**				***<0.0001***	
0	Reference				
1	1.23	1.16	1.30		***<0.0001***
2 or more	1.81	1.68	1.96		***<0.0001***
**Distance to Treatment Center**				0.4612	
Less than 10 mi	Reference				
10-25 mi	1.03	0.97	1.09		0.2945
25-50 mi	0.97	0.89	1.05		0.4030
50-100 mi	0.96	0.87	1.05		0.3564
100 mi or more	1.00	0.89	1.13		0.9676

**Zip Code Income Level**					***<0.0001***	
$63,000+	Reference					
$48,000-63,000	1.08	1.02	1.15			***0.0144***
$38,000-48,000	1.16	1.08	1.24			***<0.0001***
$0-38,000	1.18	1.09	1.28			***<0.0001***
**Diagnosis Year**					***<0.0001***	
2006 - 2008	Reference					
2009 - 2011	0.99	0.92	1.07			0.8644
2012 - 2014	0.93	0.87	1.01			0.0680
2015 - 2016	0.78	0.72	0.85			***<0.0001***
**Tumor Size**					***<0.0001***	
No Mass Found/Microscopic	Reference	Reference				
Less than 2cm	1.51	1.22	1.88			***0.0002***
2-5cm	1.73	1.38	2.16			***<0.0001***
Greater than 5cm	2.11	1.66	2.69			***<0.0001***
**Resection Margins**					***<0.0001***	
No Residual Tumor	Reference	Reference				
Residual Tumor	1.35	1.26	1.46			***<0.0001***
**Primary Site**					***<0.0001***	
Head/Neck	Reference					
Trunk	0.91	0.85	0.98			***0.0100***
Limbs	0.86	0.81	0.91			***<0.0001***
Other/NOS	0.90	0.77	1.05			0.1870
**Immune Suppression**					***<0.0001***	
Immunocompetent	Reference	Reference				
Immunosuppressed	1.25	1.25	1.12	1.38		***<0.0001***
**Positive Lymph Nodes**					***<0.0001***	
None positive	Reference					
1-3 positive	1.18	1.01	1.37			***0.0358***
4-8 positive	1.44	1.22	1.69			***<0.0001***
9 or more positive	1.81	1.46	2.24			***<0.0001***
**Facility Type**					***0.0165***	
Academic/Research Program	Reference					
Community Cancer Program	1.10	1.03	1.17			***0.0060***
Integrated Network Program	1.03	0.95	1.12			0.4664
**Facility Case Volume**					***0.0002***	
Lowest Third	Reference					
Middle Third	0.95	0.90	1.01			0.1024
Largest Third	0.84	0.78	0.92			***<0.0001***

Adj HR: Adjusted Hazard Ratio
Chemo: Chemotherapy
CI: Confidence Interval
cm: centimeter
mi: mile(s)
NOS: Not Otherwise Specified
